# Hazelnut Shells as Source of Active Ingredients: Extracts Preparation and Characterization

**DOI:** 10.3390/molecules26216607

**Published:** 2021-10-31

**Authors:** Alessandro Di Michele, Cinzia Pagano, Agnese Allegrini, Francesca Blasi, Lina Cossignani, Enrico Di Raimo, Marco Faieta, Eleonora Oliva, Paola Pittia, Sara Primavilla, Manuel Sergi, Camilla Vicino, Maurizio Ricci, Bartolomeo Schirone, Luana Perioli

**Affiliations:** 1 Department of Physics and Geology, University of Perugia, 06123 Perugia, Italy; 2 Department of Pharmaceutical Sciences, University of Perugia, 06123 Perugia, Italy; francesca.blasi@unipg.it (F.B.); lina.cossignani@unipg.it (L.C.); camivici@live.it (C.V.); maurizio.ricci@unipg.it (M.R.); luana.perioli@unipg.it (L.P.); 3 Department of Agricolture and Forestry (DAFNE), Università degli Studi della Tuscia, Via San Camillo de Lellis, 01100 Viterbo, Italy; agnese.allegrini@gmail.com (A.A.); schirone@unitus.it (B.S.); 4 Istituto Zooprofilattico dell’Umbria e delle Marche, Via G. Salvemini 1, 06126 Perugia, Italy; e.diraimo@izsum.it (E.D.R.); s.primavilla@izsum.it (S.P.); 5 Faculty of Bioscience and Agro-Food and Environmental Technology, University of Teramo, Via R. Balzarini 1, 64100 Teramo, Italy; mfaieta@unite.it (M.F.); eoliva@unite.it (E.O.); ppittia@unite.it (P.P.); msergi@unite.it (M.S.)

**Keywords:** hazelnut shells by-products, waste, valorization, extracts, maceration, ultrasonic bath, high-power ultrasonic, antioxidant activity, antimicrobial

## Abstract

Hazelnut shells represent a waste material (about 42% of the total biomass) deriving from hazelnut harvest. These are mainly used as a heating source; however, they represent an interesting source of polyphenols useful in health field. The impact on phenolic profile and concentrations of hazelnut shell extracts obtained by three extraction methods (maceration, ultrasonic bath, and high-power ultrasonic), as well as temperature, extraction time, and preventive maceration, was studied. The prepared extracts were characterized in terms of chemical composition, antioxidant and antimicrobial activities. Eighteen different phenolic compounds were identified and quantified by chemical analysis and gallic acid was the most abundant in all the extracts analyzed. Other relevant compounds were chlorogenic acid, protocatechuic acid and catechin. Preventive maceration had a positive effect on the extraction of different types of compounds regardless of the method performed. Application of the high-power ultrasonic method had different effects, either positive or negative, depending on the type of compound and extraction time. All the prepared extracts showed antioxidant activity especially those prepared by maceration, and many of them were able to inhibit the growth of both *B. subtilis* and *B. cereus*.

## 1. Introduction

*Corylus avellana* L. (hazelnuts) are widely cultivated, especially in Turkey, Italy and Spain, as kernels represent a source of many compounds, such as proteins (10–20%), fatty acids (e.g., oleic and linoleic acids), phospholipids (e.g., phosphatidylcholine), sterols (e.g., sitosterol), amino acids (e.g., arginine, leucine, glutamic acid), sugars, vitamins (vitamins B1, B2, B6, niacin, ascorbic acid, folic acid, α-tocopherol), minerals, flavonoids (quercetin, myricetin, kaempferol, eriodictyol, catechin, epicatechin, gallocatechin), phenolic acids (gallic acid, caffeic acid, *p*-coumaric acid, ferulic acid, sinapic acid) [1,2,3,4]. Hazelnut cultivars show a high level of genetic diversity for traits such as vigor, growth habits, nut size, shape and shell thickness. Cultivars most commonly used in food industry are Tonda Gentile delle Langhe (also named Tonda Gentile Trilobata), Tonda Gentile Romana, Tonda di Giffoni, S. Giovanni, Mortarella, and Riccia di Talanico, which are cultivated in Italy. Among them, Tonda di Giffoni and Tonda Gentile delle Langhe have gained the European Protected Geographical Indication (PGI) label, and they are known as “Nocciola di Giffoni” and “Nocciola Piemonte”, respectively [1]. Hazelnut skins, hard shells, green leafy covers and hazelnut tree leaves are by-products of the roasting, cracking, shelling/hulling, and harvesting processes, respectively [1].

Nowadays, hazelnut by-products and waste have a low commercial value, with the exception of hazelnut hard shells, currently used as a heating source after burning and as raw material to produce furfural in dye industry [5,6]. Hazelnut green leafy covers and tree leaves, on the other hand, are rarely used as organic fertilizers for hazelnut trees and as raw material for compost production. 

Nevertheless, in recent years, some studies have been performed in order to evaluate the potential of hazelnut by-products (skins, shells, hazelnut tree leaf) as sources of natural antioxidants and as functional food and functional ingredients useful in both health field and food industry [1,7,8]. Several phenolic compounds, for example, have been detected in hazelnut hard shells, as shown in Table 1 [3].

Waste materials deriving from hazelnut harvest represent about 42% of the total biomass. The amount of waste deriving from industrial processes has increased substantially in recent years. Many industries have started to produce different types of by-products, rich in valuable compounds to recycle. Their characterization and valorization can convert them into high-value products with applications in different fields. The possibility of extracting polyphenols from hazelnut waste, such as shells, is gaining more and more interest in industries, as it allows to solve the problem of disposal while offering a new source of active ingredients. Hazelnut shells, in fact, could represent a valuable source of molecules to be used as active ingredients in nutraceuticals, dietary supplements, medical devices, pharmaceutical products or cosmetics [2]. 

Both antioxidant and radical scavenging activities of the phenolic compounds obtained from *C. avellana* by-products [1,9,10] depend on the extraction method as well as the source (shells, green leafy covers, flowers, and leaves). It is interesting to note that despite kernels (the edible part of hazelnuts) being rich in polyphenols, literature data demonstrated that extracts obtained from *C. avellana* by-product shells, green leafy covers, flowers and leaves exhibited stronger activities than hazelnut kernels [1]. These findings suggest that a deep investigation of hazelnut by-products’ potential applications in the health field is necessary. The wide range of phytochemicals and phenolic compounds contained possess substantial antioxidant and radical scavenging activities, as well as anticarcinogenic, antimutagenic, and antiproliferative effects [2].

In this study the attention was focused on hazelnut shells, notoriously considered a waste material. The aim of this work was the valorization of hazelnut waste, in particular hazelnut shells, demonstrating their suitability as a source of active ingredients to be used in health field, offering a concrete solution to the problem of their disposal. 

With this in mind, the research has the objective to prepare extracts from hazelnut shells and to perform a deep characterization of both the qualitative and quantitative compositions as well as the biological activities in order to find possible applications in health field. In order to reach this objective, three different extraction methods were investigated: (i) maceration (MAC), (ii) ultrasonic bath (UB) and (iii) high-power ultrasonic (HPU); different extraction times were also investigated. The obtained extracts were deeply characterized in terms of chemical composition, antioxidant, radical scavenging and antimicrobial activities. The proposed approach could represent an interesting research target, with environmental and economic benefits, promoting the development of the circular bioeconomy [11]. 

Hazelnut shells are inexpensive and bio-sustainable, representing a valid alternative to the plant-derived extracts commonly used in both cosmetic and pharmaceutical formulations. Moreover, when waste products come from organic farming, they certainly represent an even more valuable source of safe extracts. They in fact, lack any residual pesticides or potentially toxic chemicals [12].

## 2. Results and Discussions

### 2.1. Hazelnut Shell Grinding and Sieving

Hazelnuts were washed with tap water, air-dried, and hand-crushed before the extraction. Obtained shells were ground in order to obtain a product with high surface area to be exposed to the extraction solvent. This factor, together with the extraction conditions (temperature and stirring) and the extraction solvent, influences the qualitative and quantitative composition of the final extract. Hazelnut shells were ground by a knife mill and then sieved (for details, see 3.2. Method section).

### 2.2. Extracts Preparation

Three extraction methods were used for the extraction (as described in the Method section): 

(A) Maceration (MAC), 

(B) Ultrasonic Bath (UB), 

(C) High-Power Ultrasonic (HPU). 

Maceration is the classical method already used with success for other vegetal matrices [13,14,15] and for this reason chosen for comparison. 

As this method requires energetic expenditure, alternative and efficient methods useful to obtain hazelnut extract with limited energy dissipation have been explored.

Recently, the demand for efficient and economical extraction processes has been increasing, especially from the food, chemical, and pharmaceutical industries. In response to this, many extraction techniques have been developed, especially for the extraction of phenolic compounds from vegetable materials, such as ultrasound-assisted extraction (either with a bath or with a higher power probe), supercritical fluid extraction and microwave-assisted extraction [16]. Ultrasound-assisted extraction is more efficient than traditional extraction methods in terms of reduced extraction time, extraction solvent volumes, extraction temperatures, and high yield values. On the other hand, it does not allow selective extraction, because it results in the total escape of all the molecules contained in the plant material, regardless of affinity with the solvent used. The good extraction yield using ultrasound-assisted extraction techniques is attributable to the acoustic cavitation and mechanical effects, able to disrupt cell walls, reduce particle size and enhance mass transfer across cell membranes. It is important to underline that all these phenomena can only occur in the presence of solvent. This extractive technique is “bio accredited” because it is based only on a physical process in which chemical adjuvants are not necessary. Solvents such as water and alcohol are used, regardless of the solubility of the active ingredients [16].

The extraction conditions, as well as the shell powder size, were defined according to the following observations made during preliminary evaluations and considerations:

(i) particles of size < 500 μm make the filtration process difficult; thus, particles with higher dimensions were selected; (ii) the solid/liquid ratio (2 g/100 mL) and the extraction time were chosen according to previous studies based on the extraction of active ingredients from vegetal matrices [13,14]; (iii) EtOH was used as extraction solvent because it is a green solvent reusable after distillation.

Samples of hazelnut shells were prepared with and without preventive maceration overnight in the extraction solvent (static conditions) in order to evaluate the impact of the contact time on the final yield. 

The yield (% *w*/*w*) after extracts freeze-drying was calculated for each extract and is reported in Table 2.

Among the selected methods, MAC’s extraction efficiency, in terms of final yield, is particularly influenced by both contact times and temperature. This could be particularly true for fibrous matrices such as hazelnut shells. For this reason, two different temperatures and different contact times were assayed for MAC. Comparing the samples obtained at 25 °C, it is clearly detectable that the increase of the contact time from 60 min to 180 min promotes an increase of the final yield. Increasing the temperature to 45 °C it is possible to observe an increase of the final yield comparing the same times (e.g., A vs E and C vs G). As further control, taking into account the fibrous nature of hazelnut shells, extracts (I, J) were prepared working at 45 °C and prolonging the contact time to 300 min. It was hypothesized that a prolonged contact time could favor a better penetration of the extraction solvent into the shells with consequent improvement of the yield, effectively observed in the final extracts, compared to that obtained using the same contact times (e.g., J vs H).

In all the extractions performed by MAC preventive maceration allowed us to obtain a better yield value.

In the case of UB, three different contact times were assayed: 60, 120, and 180 min. The highest time assayed for MAC (namely 300 min) was not investigated in this case, as it was considered very long and energy consuming for this technique. Increasing the sonication time it is possible to observe an increase of the final yield in samples both with and without preventive maceration. 

In the case of HPU it can be noted that the yields of the extracts prepared with preventive maceration, using 5 min and 60 min as extraction times, show the same yield, suggesting that in the case of this technique, the increase of the extraction time does not positively influence this aspect. 

Considering the extraction time of 60 min, assayed for all the techniques (MAC, UB, and HPU), HPU allowed to obtain the highest yield compared to MAC and UB. Certainly, the samples obtained by MAC for prolonged contact times and at 45°C (sample J) produced the highest yield (1.42%); however, some considerations must be noted. MAC is a very simple extraction technique consisting in putting the material in contact with the chosen solvent for a suitable time [17]. Phenolic compounds are traditionally extracted by long maceration and at heat reflux. These extraction techniques are laborious, time consuming and require large amounts of organic solvents [16]. 

Specific literature data about the ultrasound-assisted extraction process (such as temperature, frequency, power, solvent type, and solvent-to-material ratio effects on extracted compounds and their functionality) are poor. In general, it is reported that 1) high extraction temperatures (> 50°C) degrade polyphenols, 2) the lowest frequencies within the ultrasound power range below 40 kHz are most effective, 3) polyphenol yield generally increases as power increases but with a threshold beyond which no significant increase is observed, 4) higher ultrasound power can produce free hydroxyl radicals which degrade polyphenols, especially in the presence of high water content. Furthermore, it does not only contribute to increased extraction yield in terms of polyphenol content, but can also preserve and increase the biological activity of polyphenol extracts, compared to traditional maceration products [18].

It is interesting to highlight that HPU allowed us to obtain a final yield of 0.96 using an extraction time of 5 min (sample R). In the case of MAC, a similar value is obtained working at 45 °C for 180 min (sample H). 

### 2.3. Total Phenol Content and Antioxidant Activity

The prepared extracts (Table 2) were firstly submitted to analyses in order to evaluate their antioxidant activity. Folin–Ciocalteu method was used to evaluate the TPC of hazelnut shell extracts, while ABTS, DPPH, and FRAP assays were used to measure the antiradical (ABTS and DPPH) and reducing (FRAP) activities, respectively. 

Table 3 shows the values of TPC and antioxidant activity evaluated by ABTS, DPPH, and FRAP.

Taking into consideration the UB method, the best TPC values were obtained with an extraction time of 180 min, without a particular improvement when preventive maceration was used; considering the HPU method, the longer time (60 min) gave the best results, also without pretreatment. Generally, higher TPC values were observed with increasing temperature for both UB and HPU methods. When the classical maceration was carried out at the same temperature of UB and HPU, the best TPC values were obtained after 60 min with preventive maceration. Moreover, it can be observed that both time and temperature parameters had a positive impact on TPC value using classical maceration; in fact, the highest value was obtained at 45 °C for 300 min. However, it has been reported that more drastic extraction conditions could promote a possible partial degradation of phenolic compounds [19].

Generally, it can be observed that preventive maceration allowed us to obtain higher values only in some cases. Considering the UB method, comparable or slightly higher TPC values were obtained after 60, 120 or 180 min. Considering the HPU method, preventive maceration was useful when the extraction times were 5 or 30 min. 

Similar TPC values (about 3.5–12 mg GAE/g) were reported by Yuan et al. [20]. They observed a wide range of variability; in fact, TPC value of the extracts significantly increased with the reduction of the particle size of hazelnut shells at all extraction times (2–12 h). These authors found that higher amounts of TPC were obtained by 50% aqueous solution compared with 20% and 80% aqueous solutions for all three solvents (acetone, methanol, ethanol). Masullo et al. [21] reported a higher value of TPC (340.44 μg GAE/mg) for methanol hazelnut (Nocciola di Giffoni PGI) shell extracts. In comparison with conventional solvent extraction, Pérez-Armada et al. [22] recently reported the highest value of TPC (3.37 g GAE/100 g) for hazelnut shell extracts obtained by autohydrolysis processes and recovered using resins.

In order to evaluate the antioxidant activity of extracts, three complementary spectrophotometric in vitro assays were carried out. As is widely known, the ABTS assay measures the ability of antioxidants to scavenge ABTS free radical by electron donation, while DPPH assay evaluates the ability of antioxidants to scavenge the chromogen DPPH free radical. On the base of previous considerations on TPC values, the best ABTS and DPPH values were obtained using UB method for 180 min and HPU method for 60 min without pretreatment. The reducing power of extracts was evaluated by FRAP assay, and trends similar to those already discussed for TPC and ABTS-DPPH can be observed. In regard to antioxidant assay data (ABTS, DPPH, FRAP), only in some cases was the previous overnight maceration useful to improve these parameters, while in other cases, the values are similar between them or even lower.

As already observed for yield values, taking into consideration the extraction time of 60 min, HPU allowed us to obtain the highest value of ABTS, DPPH, and FRAP compared to UB. It should be further observed that since both ultrasound-based techniques were carried out at 25 °C, it could be assumed that there are no negative effects on the antioxidant activity of the bioactive compounds [19].

Literature data about the nutritional composition and antioxidant activity of hazelnut shells (US-grown cultivars) [23] report ABTS values from 35.7 μmol TE/g (Nebraska hybrid cultivar) to 169.7 (Oregon cultivar) μmol TE/g. A wide range of FRAP values variability (30–110 μmol TE/g) was also reported by Yuan et al. [20].

**Figure 1:**
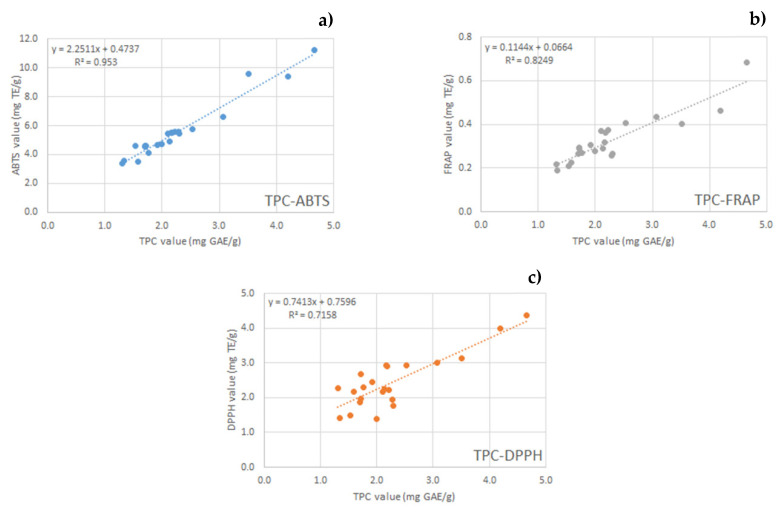
Correlations between TPC and antioxidant activity assays (ABTS, FRAP, DPPH) of all extracts. Correlation between TPC and ABTS (**a**); correlation between TPC and FRAP (**b**); correlation between TPC and DPPH (**c**).

Interestingly, TPC values were significantly and positively correlated with ABTS (R² = 0.9530), FRAP (R² = 0.8249), and DPPH (R² = 0.7158) values, suggesting that antioxidant properties of hazelnut shell extracts are attributable to polyphenols (Figure 1). The lower R^2^ value (TPC vs DPPH) could be due to low-molecular-weight antioxidants being more effective as DPPH^•^ scavengers, while high-molecular-weight bioactives (polymers) may be ineffective against this radical, as reported also by Paixão et al. [24].

**Figure 2:**
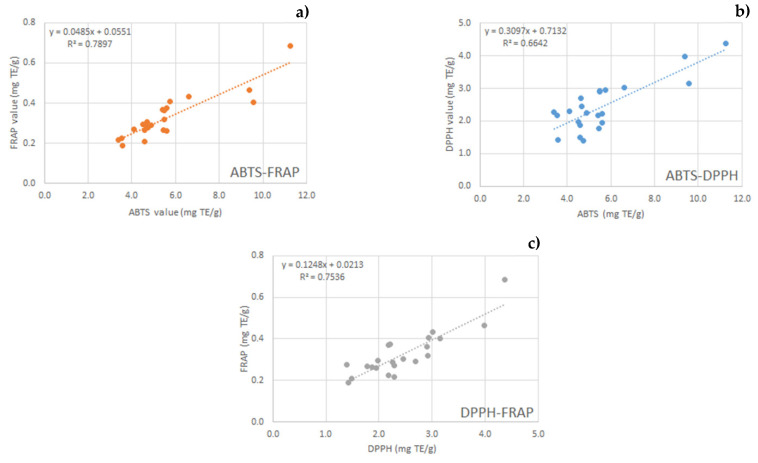
Correlations between values of antioxidant activity assays (ABTS, DPPH, FRAP) of all extracts. Correlation between ABTS and FRAP (**a**); correlation between ABTS Correlation between TPC and ABTS (**a**); correlation between TPC and FRAP (**b**); correlation between TPC and DPPH (**c**). DPPH (**b**); correlation between DPPH and FRAP (**c**).

Moreover, an interesting correlation study was also carried out among the three antioxidant assay parameters, and high correlation values (R^2^ = 0.7897 ABTS vs FRAP; R^2^ = 0.7536 DPPH vs FRAP; R^2^ = 0.6642 ABTS vs DPPH) were found (Figure 2). Xu et al. (2012) also reported that TPC and ABTS values were significantly and positively correlated with bioactive hazelnut shell compounds, while no significant correlation was found between DPPH and phenolic compound.

The observed antioxidant activity is very interesting and makes the extract an interesting material to be used in both the pharmaceutical and cosmetic fields.

In the case of wounds, for example, the release of pro-inflammatory mediators as well as radical oxygen species occurs [25]. Thus, treatment with molecules with antioxidant activity could be useful in the suppression of factors responsible for healing delay. 

### 2.4. Chemical Analysis

Eighteen different phenolic compounds were identified and quantified in the shell extracts through HPLC–MS/MS analysis (Table 4). 

Among the phenolic compounds analyzed, gallic acid was the most abundant, representing up to 89% of the phenolic fraction identified, with a concentration ranging, depending on the type of extraction performed, from 722.0 to 12940.0 μg per gram of extract. Shahidi et al. 2007 [2] reported gallic acid as the most abundant phenolic compound in hazelnut hard shell extract at a concentration of 3261.0 μg/g within the range of values observed in this study. Moreover, Ciemniewska-Zytkiewicz et al. in 2015 [4] also reported gallic acid as the first phenolic compound in hazelnut hard shell extracts, but at significantly lower concentrations than the ones found in this study. Differences in phenolic compounds concentrations may arise from the different extraction techniques and conditions, types of solvents used, or origin of the samples [1,26]. 

Yuan et al. [20] reported a concentration of gallic acid of 62.17 μg per gram of shell, in agreement with the concentrations (based on extraction yield) observed in this study; however, other phenolic compounds were reported to be present at higher concentrations, with catechin being the most abundant. 

Catechin was the second phenolic compound in all extracts obtained through UB and HPU, and among the most abundant phenolic compounds in MAC extracts, with a maximum concentration of 2760.0 μg per gram of extract observed in the sample macerated at 45 °C for 300 min. 

Other major polyphenols found in the extracts were chlorogenic acid, rutin, and protocatechuic acid at a maximum concentration of 1345.5, 2611.0, and 1794.0 μg per gram of extract, respectively. 

Protocatechuic acid, catechin, and gallic acid were also reported as the most abundant compounds in the phenolic profile of hazelnut skin [9], but in amounts significantly lower than in hazelnut shell, in agreement with the composition of hazelnut by-products previously described [2]. 

This is the first time that caffeic acid, epigallochatechin gallate, syringic acid, isoquercetin, tyrosol, ferulic acid, luteolin, apigenin, diosmetin, and naringenin have been identified in hazelnut shells. 

The different process variables (i.e., extraction techniques, time, temperature and static preventive maceration) had an impact on the phenolic profile and concentrations in the different extracts.

Preventive static maceration had a positive impact on the extraction of catechin for both UB and HPU extracts, which showed higher concentration of the flavanol compound compared to the analogous extracts which weren’t subject to preventive maceration. Chlorogenic acid concentration in UB extracts was positively affected by preventive static maceration as well, whereas, in HPU, the longest time of extraction was detrimental for chlorogenic acid. The exposure to HPU for 60 min may have induced a partial degradation of the phenolic acid, with extracts subject to 30 min HPU showing higher concentration in chlorogenic acid. In the equivalent samples which underwent preventive maceration, the phenomenon was more marked. Long extraction time can have a negative effect on some phenolic compounds, triggering oxidation phenomena and reduction of the content [27].

In regard to the extraction methods samples obtained through UB, they showed a higher concentration of gallic acid, chlorogenic acid, and catechin compared to HPU and MAC, whereas no relevant effects were observed on specific phenolic compounds using the HPU extraction method. MAC had a positive effect on the extraction of epigallocatechin gallate, syringic acid, rutin, isoquercetin, 3-OH benzoic acid, *p*-coumaric acid, ferulic acid, lutelolin, quercetin, apigenin, diosmetin, kampferol, and protocatechuic acid; however, no significant effect of temperature, time, or the combination of both seemed to outline when this method was used. Regarding gallic acid, the most abundant phenolic compound, MAC, was less effective in the extraction compared to HPU and UB methods, with the exception of the two extractions performed for the longest times (i.e., 300 min). 

All these compounds here identified and determined should significantly contribute to the antioxidant ability of the hazelnut shell extracts. 

### 2.5. Antimicrobial Activity

The antimicrobial activity of the prepared extracts (Table 2) was assayed against the strains reported in Table 5 using water solutions of each extract at the concentration of 10 mg/mL. In the case of *B. subtilis,* the concentrations 5 mg/mL and 2.5 mg/mL were assayed as well. After incubation, the inhibition halo was measured for each strain tested, and the obtained results are reported in Table 5.

**Figure 3:**
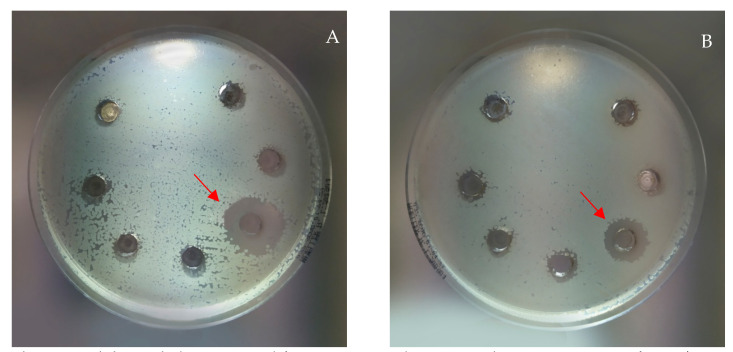
Inhibition halos measured for extract K (red arrow) at the concentrations of 5 mg/mL, 15 mm (**A**), and 2.5 mg/mL, 13 mm (**B**).

The extracts revealed antimicrobial activity only against some of the Gram-positive bacteria tested (*B. subtilis*, *B. cereus*, *S. aureus,* and *S. epidermidis*), while Gram-negative bacteria and yeast were not sensitive to all the assayed extracts. The obtained results are in accordance with those obtained by other authors with aqueous extracts of hazelnut kernels [28]. *B. subtilis* was the most susceptible bacteria, with extracts A–M active at the concentration of 10 mg/mL and extract K, also active at the two lowest concentrations tested (Figure 3). This antimicrobial activity, in particular against *B. subtilis*, could be mainly attributable to the high content of gallic acid measured for these extracts (Table 3), as already observed by other authors [29]. Due to the high number of phenolic molecules in each extract, it is not possible to attribute the observed activity to a specific molecule. It could certainly be hypothesized that it is attributable to a synergistic effect of the most abundant molecules in each extract. 

Phenolic compounds’ antimicrobial capacity is well known [30] and can have concrete applications in food production in order to replace the use of other, less safe compounds to inhibit or limit microbial growth of pathogenic and spoilage microorganisms [31,32]. It is interesting to note that the prepared extracts, properly formulated, could have interesting applications in health field, for example in the treatment of wound infections due to those bacteria sensitive to the extracts. *B. subtilis*, for example, is frequently responsible for biofilm formation, often associated with hospital-acquired infections [33]. *S. aureus* and *S. epidermidis* are among the main bacteria responsible for wound infections [34], and are also responsible for biofilm formation [35]. It is interesting to underline that *S. aureus* is labelled among multi-drug-resistant strains [36], for which antimicrobial therapies alternative to the conventional ones are necessary.

The inhibition of *B. cereus* is also very important in the treatment of wounds, because this is involved in many skin infections and postoperative and post-traumatic wound infection [37].

## 3. Materials and Methods

### 3.1. Reagents

Hazelnuts (cultivar Tonda Gentile Romana) were supplied by Fattoria Lucciano Soc. Agr. s.s. (Civita Castellana, Viterbo, Italy).

Ethanol 96% (EtOH), Folin–Ciocalteu reagent, sodium carbonate (Na_2_CO_3_), gallic acid (GA), 2,2-diphenyl-1-picrylhydrazyl (DPPH), (±) 6-hydroxy-2,5,7,8-tetramethylchromane-2-carboxylic acid (Trolox, 97%), 2,2’-azino-bis(3-ethylbenzothiazoline-6-sulphonic acid (ABTS), potassium peroxodisulfate (K_2_S_2_O_8_), sodium acetate trihydrate (CH_3_COONa·3·H_2_O), acetic acid (CH_3_COOH), 2,4,6-tri-(2-pyridyl)-s-triazine (TPTZ), hydrochloric acid (HCl), ferric chloride (FeCl_3_), ferrous sulphate heptahydrate (FeSO_4_·7·H_2_O), methanol (MeOH), acetic acid (CH_3_COOH), acetonitrile (ACN), polypropylene glycol (PPG), the standards of selected phenolic compounds gallic acid, protocatechuic acid, (-)-epigallocatechin gallate, 3- OH-benzoic acid, chlorogenic acid, caffeic acid, catechin, syringic acid, rutin, *p*-coumaric acid, isoquercetin, ferulic acid, luteolin, quercetin, naringenin, apigenin, diosmetin, and kaempferol were purchased from Sigma Aldrich (Milano, Italy).

Ultrapure water was obtained by reverse osmosis process in a MilliQ Millipore system (Roma, Italy). Calcium chloride (CaCl_2_) was purchased from Carlo Erba (Milano, Italy).

Brain Heart Infusion (BHI) broth: dehydrated beef heart infusion 250 g/L, dehydrated beef brain infusion 200 g/L, disodium phosphate 2.5 g/L, sodium chloride 5 g/L, glucose 2 g/L, proteose peptone 10 g/L (Biolife Italiana s.r.l., Monza, Milano, Italy) in deionized water, pH 7.3 ± 0.2, 25°C. Mueller Hinton Agar (MHA): 2.0 g beef extract, 17.5 g casein hydrolysate, 1.5 g starch, 17.0 g agar (Oxoid Limited, Basingstoke, United Kingdom) in deionized water, pH 7.3 ± 0.2, 25°C. Mueller Hinton Agar 5% sheep blood (MHAB): Mueller Hinton Agar (Oxoid Limited, Basingstoke, United Kingdom), defibrinate sheep blood (Blood Farms of Fiastra Maddalena, Teramo, Italy) in deionized water, pH 7.3 ± 0.2, 25°C.

### 3.2. Hazelnut Shells Grinding and Sieving

Hazelnuts were hand-crushed, and then the shells were ground by the knife mill GRINDOMIX GM 200 (Retsch, Predengo, Cremona, Italy) working at 4000 rpm, for 2.30 min. The obtained ground powder was sieved by steel sieves (Endecotts Ltd., London, UK) with mesh sizes of 4.760 mm, 2.38 mm, 1 mm, 710 μm, 500 μm, and 400 μm. 

### 3.3. Extracts Preparation 

The extraction was performed using 2 g of ground hazelnut shells (1 g with of dimension 710–1000 μm and 1 g of dimension 500–710 μm). Three different extraction procedures were performed using EtOH 70% as extraction solvent (100 mL): 

A) maceration (MAC) at 25 °C and 45°C, at ordinary pressure, under magnetic stirring (1500 rpm);

B) by using ultrasonic bath (UB) RK 100H (BANDELIN SONOREX, Berlin, Germany), power of 80/320 W and a frequency of 35 KHz, working at 25 °C for different times (5 min ON and 10 min OFF); 

C) high-power ultrasonic (HPU) by an emitted power of 750 W and transmitted power of 200 W, frequency of 20 KHz, amplitude of 50%, working at 25 °C, for different times using Horn Type ultrasonic probe VCX750 (SONICS, Newtown, CT, USA). 

The extraction procedures were carried out with and without preventive static maceration overnight at room temperature (R.T.) of the ground hazelnut shells (2 g) in the extraction solvent (100 mL). 

After each extraction, the suspension obtained was filtered under vacuum by a cellulose membrane Whatman 41 filter (Whatman GmbH, Dassel, Germany). The solvent was evaporated by rotary evaporation (R-100, BUCHI, Cornaredo Italy) at 35 °C. The solid obtained was dissolved in 5 mL of bidistilled water and freeze-dried (DRYWINNER, Heto, Gydevang, Denmark). The products obtained were stored in a desiccator under CaCl₂ until use. The yield of each extraction was calculated according to Equation (1):

(1) $$\mathrm{yield}\;\%=\frac{\mathrm{weight}\;\mathrm{of}\;\mathrm{shell}\;\mathrm{hazelnut}\;}{\mathrm{weight}\;\mathrm{of}\;\mathrm{freeze}\;-\;\mathrm{dryed}\;\mathrm{extract}}\;\times \;100\;$$

### 3.4. Total Phenol Content and Antioxidant Activity

The total phenol content (TPC) was determined by a spectrophotometric method, according to the Folin–Ciocalteu procedure, modified by Pagano et al. [38]. Deionized water, 20% Na_2_CO_3_ solution, and Folin and Ciocalteu’s reagent were added to an aliquot of the diluted extracts and kept protected from light for 30 min; then, the absorbance at 750 nm was measured (Lambda 20 spectrophotometer, PerkinElmer, Inc; Waltham, MA, USA). A calibration curve was prepared using gallic acid solutions, and the results were expressed as mg of gallic acid equivalents (GAE) per gram of hazelnut shells (mg GAE/g). 

The radical scavenging activity of the extracts was measured by ABTS and DPPH assays. ABTS assay was carried out following the procedure described by Rocchetti et al. [39]. Sample was added to ABTS^+ •^ reagent and, after 10 min, the absorbance was measured at 734 nm. A calibration curve was prepared using Trolox solutions, and the results were expressed as mg Trolox equivalents (TE) per gram of hazelnut shells (mg TE/g). The DPPH assay was carried out according to the procedure reported by Ianni et al. [40]. DPPH (0.06 mM in ethanol) was added to the sample, and the mixture was kept in the dark for 30 min, after which the absorbance at 517 nm was measured. A calibration curve was prepared using Trolox solutions, and the results were expressed as mg TE/g.

The reducing power of the extracts was determined by FRAP (ferric reducing antioxidant power) assay. The FRAP reagent, prepared by mixing acetate buffer, TPTZ, and FeCl_3_·6·H_2_O, was added to the extracts, and then the samples were left in the dark for 30 min. The absorbance of the sample was measured at 593 nm. A calibration curve was prepared using Trolox solutions, and the results were expressed as mg TE/g.

All spectrophotometric determinations were carried out in duplicate. Results of TPC and antioxidant activity were reported as mean value ± standard deviation (SD).

### 3.5. Chemical Analysis

The analysis of the phenolic composition was performed according to the method described by Oliva et al. in 2021 [41]. Briefly, one mg of sample was dissolved in 1 mL of solution water–methanol (90/10 *v*/*v*) at pH 3.00. The clean-up phase was carried out through SPE technique, by using a Strata-XL 100 µm Polymeric Reversed Phase (Phenomenex), and 10 μL of sample was loaded onto the cartridge and eluted in methanol. Afterwards, elutes were diluted (1:100) before being injected.

The chromatographic analysis was performed by an UHPLC system Nexera XR from Shimadzu (Tokyo, Japan). The analytes were separated by using an ACE Excel 2 C18-PFP column (10 cm × 2.1 mm id) from ACE (Aberdeen, UK) packed with particles of 2 μm. A column saver was also used to protect the column from damaging contaminants and microparticulate. The flow rate was 300 μL/min, driven completely into the MS ion source. The mobile phases were:

A phase: 99% H_2_O, 1% CH_3_COOH;

B phase: 100% acetonitrile.

The elution of the analytes was carried out with a gradient structured according to the following steps:

1. Linear increase of B phase from 5% to 30% in 3.0 min;
2. Linear increase of B phase from 30% to 40% in 1.4 min;
3. Linear increase of B phase from 40% to 60% in 2.5 min;
4. Linear increase of B phase from 60% to 99% in 1.5 min;
5. Isocratic of B phase at 99% for 0.5 min.

The separation of the analytes takes place in 10 min. The total duration of the analysis is 12 min, including the 2 min of rebalancing of the initial conditions between one analysis and the next.

For the identification of the analytes, the UHPLC system was coupled with a QTRAP 4500 tandem mass spectrometer (Sciex, Toronto, Canada), equipped with a heated electrospray ionization source (V-source), and operated in the negative ionization mode.

### 3.6. Antimicrobial Activity

The experiments were performed using the agar well diffusion technique [42] on the strains reported in Table 6. The stored strains were revitalized on BHI Broth and incubated according to the growth conditions shown in Table 6.

An initial suspension of 0.5 McFarland in 0.9% sterile saline solution was prepared for each organism, and 100 µL was distributed on MHA plates with a swab, except for *S. pyogenes,* where MHAB plates were used. At the time of use, extracts were suspended with sterile demineralized water to obtain the concentration of 10 mg/mL, and in the case of *B. subtilis,* concentrations of 5 mg/mL and 2.5 mg/mL were also tested. Then, 50 μL of extract suspension was placed in a hole (diameter of 7 mm) previously made in the center of the plates, and then incubated according to the growth conditions shown in Table 6. For each bacterial strain, each extract suspension was tested. At the end of the incubation, the presence and the diameter of the inhibition halo was measured by a gauge.

## 4. Conclusions

The aim of this work was to reuse a waste material, the hazelnut shells, as a source of active ingredients to be used in the health field. 

The effect of three extraction methods (maceration, ultrasonic bath, and high-power ultrasound), as well as contact time, temperature, and preventive maceration, on the qualitative and quantitative composition of the extracts was studied. Gallic acid was the most abundant phenolic compound, in agreement with data reported in previous studies. Other major polyphenols found in the extracts were chlorogenic acid, rutin, and protocatechuic acid with the highest concentrations measured being 1345.5 μg/g, 2611.0 μg/g, and 1794.0 μg/g respectively. In general, preventive maceration favored the extraction of different phenolic compounds such as catechins. Based on the obtained results, the choice of the most suitable extraction method and process variables must be performed depending on the type of compound of interest. Further studies on this topic could provide valuable information for optimal extraction conditions.

In vitro studies highlighted that the prepared extracts show interesting antioxidant activities, especially in the extracts obtained by the maceration method. This activity is very interesting as it could be exploited in both the pharmaceutical and cosmetic fields, for example, in the treatment of wounds in which the healing process is often delayed by the presence of radical oxygen species. Moreover, many of the prepared extracts exhibit antimicrobial activity, especially against *B. subtilis* and *B. cereus*.

Moreover, the purposed extraction methods are eco-friendly and sustainable, favoring the reuse of a waste material and respecting the environment.

## Figures and Tables

**Table 1:** Phenolic compounds in hazelnut shell.

Phenolic Compounds	More Abundant Components
Phenolic Acids	gallic acid, vanillic acid, methyl gallate, veratric acid, galloylquinic acid, coumaroyl acid, quinic acid, feruloylquinic acid, protocatechuic acid
Flavonoids	quercetin, myricetin, quercetin 3-rhamnoside, myricetin 3-rhamnoside, rutin, taxifolin, naringin, catechin, epicatechin, epigallocatechin
Tannins	four isomers of B-type procyanidin
Diaryleptanoids	giffonin V, giffonin P, carpinontriol B
Lignans	ficusal, erythro-(7S,8R)-guaiacylglycerol-β-O-4′-dihydroconiferyl alcohol, erythro-(7S,8R)-guaiacylglycerol-β-coniferyl aldehyde ether, erythro-(7R,8S)-guaiacylglycerol-β-O-4′-dihydroconiferyl alcohol, dihydrodehydrodiconiferyl alcohol, balanophonin

**Table 2:** Extraction conditions and yields.

Extract	Extraction Method	Extraction Temperature (°C)	Extraction Time (min)	Preventive Maceration	Yield of Freeze-Dried (%*w*/*w*) ± SD
**A**	MAC	25	60	no	0.32 ± 0.02
**B**	MAC	25	60	yes	0.60 ± 0.03
**C**	MAC	25	180	no	0.51 ± 0.06
**D**	MAC	25	180	yes	0.64 ± 0.01
**E**	MAC	45	60	no	0.68 ± 0.03
**F**	MAC	45	60	yes	0.70 ± 0.06
**G**	MAC	45	180	no	0.81 ± 0.03
**H**	MAC	45	180	yes	1.10 ± 0.02
**I**	MAC	45	300	no	0.88 ± 0.05
**J**	MAC	45	300	yes	1.42 ± 0.07
**K**	UB	25	60	no	0.37 ± 0.19
**L**	UB	25	60	yes	0.50 ± 0.15
**M**	UB	25	120	no	0.57 ± 0.13
**N**	UB	25	120	yes	0.72 ± 0.15
**O**	UB	25	180	no	0.64 ± 0.07
**P**	UB	25	180	yes	0.77 ± 0.03
**Q**	HPU	25	5	no	0.47 ± 0.04
**R**	HPU	25	5	yes	0.96 ± 0.06
**S**	HPU	25	30	no	0.80 ± 0.22
**T**	HPU	25	30	yes	0.82 ± 0.27
**U**	HPU	25	60	no	0.96 ± 0.03
**V**	HPU	25	60	yes	0.94 ± 0.21

MAC: maceration; UB: ultrasonic bath; HPU: high-power ultrasonic; SD: standard deviation.

**Table 3:** TPC and antioxidant activity (ABTS, DPPH, and FRAP) values of hazelnut shell extracts (mean values ± SD).

Sample	TPC mg GAE/g	ABTS mg TE/g	DPPH mg TE/g	FRAP mg TE/g
**A**	1.34 ± 0.03	3.57 ± 0.07	1.42 ± 0.09	0.19 ± 0.00
**B**	2.30 ± 0.07	5.46 ± 0.14	1.78 ± 0.14	0.27 ± 0.0
**C**	1.54 ± 0.05	4.59 ± 0.14	1.48 ± 0.01	0.21 ± 0.00
**D**	1.99 ± 0.01	4.73 ± 0.13	1.22 ± 0.26	0.28 ± 0.00
**E**	3.51 ± 0.24	9.57 ± 0.18	3.14 ± 0.01	0.40 ± 0.01
**F**	2.14 ± 0.05	4.89 ± 0.08	2.25 ± 0.03	0.29 ± 0.00
**G**	4.20 ± 0.05	9.37 ± 0.01	3.99 ± 0.29	0.46 ± 0.02
**H**	2.28 ± 0.04	5.59 ± 0.11	1.95 ± 0.16	0.26 ± 0.00
**I**	3.07 ± 0.04	6.61 ± 0.20	3.02 ± 0.15	0.43 ± 0.00
**J**	4.66 ± 0.08	11.25 ± 0.34	4.37 ± 0.35	0.68 ± 0.01
**K**	1.70 ± 0.06	4.59 ± 0.43	1.86 ± 0.13	0.26 ± 0.00
**L**	1.77 ± 0.01	4.10 ± 0.09	2.29 ± 0.10	0.27 ± 0.01
**M**	1.58 ± 0.04	3.54 ± 0.00	2.18 ± 0.16	0.22 ± 0.00
**N**	1.92 ± 0.06	4.68 ± 0.25	2.45 ± 0.19	0.30 ± 0.01
**O**	2.18 ± 0.06	5.50 ± 0.03	2.91 ± 0.09	0.36 ± 0.01
**P**	2.16 ± 0.05	5.49 ± 0.22	2.92 ± 0.07	0.32 ± 0.01
**Q**	1.31 ± 0.04	3.39 ± 0.10	2.28 ± 0.14	0.22 ± 0.01
**R**	1.71 ±0.00	4.51 ± 0.08	1.98 ± 0.02	0.30 ± 0.00
**S**	1.72 ± 0.03	4.63 ± 0.33	2.69 ± 0.04	0.29 ± 0.00
**T**	2.10 ± 0.05	5.43 ± 0.05	2.18 ± 0.09	0.37 ± 0.00
**U**	2.53 ± 0.03	5.74 ± 0.13	2.94 ± 0.09	0.41 ± 0.00
**V**	2.22 ± 0.04	5.59 ± 0.06	2.22 ± 0.07	0.37 ± 0.00

ABTS, 2,2′-azino-bis(3-ethylbenzothiazoline-6-sulfonic acid) diammonium salt; DPPH, 2,2-diphenyl-1-picrylhydrazyl; FRAP, ferric reducing antioxidant power; TPC, total phenol content; GAE, gallic acid equivalents; TE, trolox equivalents.

**Table 4:** Phenolic compounds identified and quantified in hazelnut shell extracts. Data are reported as μg/g.

Sample	Gallic Acid	Chlorogenic Acid	Catechin	Caffeic Acid	EGCG	Syringic Acid	Rutin	Isoquercetin	3-OH-Benzoic Acid
**A**	2022.0 ± 319.2	317.7 ± 47.4	688.1 ± 135.7	3.4 ± 0.5	44.8 ± 5.2	108.5 ± 11.0	1897.0 ± 211.8	76.14 ± 11.9	173.6 ± 22.0
**B**	2683.0 ± 495.6	348.2 ± 44.5	1267.5 ± 234.2	4.7 ± 0.5	81.9 ± 8.7	118.9 ± 16.3	1343.5 ± 165.4	72.87 ± 16.4	451.6 ± 70.0
**C**	1797.0 ± 346.6	354.2 ± 79.9	1067.0 ± 123.1	9.2 ± 1.0	142.1 ± 15.8	127.5 ± 12.8	2611.0 ± 261.8	111.4 ± 11.8	150.3 ± 17.8
**D**	1708.0 ± 245.4	366.5 ± 45.5	547.1 ± 90.0	2.7 ± 0.3	71.9 ± 10.6	121.7 ± 18.3	1223.5 ± 153.9	229.2 ± 26.0	143.4 ± 21.8
**E**	2334.0 ± 321.6	327.6 ± 53.8	1644.0 ± 226.9	9.4 ± 1.1	328.3 ± 11.3	209.0 ± 21.7	1442.5 ± 281.2	29.35 ± 4.4	35.56 ± 5.8
**F**	2201.5 ± 231.2	377.2 ± 87.2	1133.0 ± 195.1	1.1 ± 0.1	33.0 ± 5.7	135.1 ± 17.8	226.7 ± 58.2	107.6 ± 11.5	170.8 ± 20.4
**G**	1771.0 ± 294.2	351.9 ± 35.5	894.4 ± 101.8	4.9 ± 0.6	108.4 ± 8.1	125.2 ± 12.8	715.8 ± 133.8	19.04 ± 2.4	109.1 ± 11.4
**H**	1958.5 ± 356.4	394.2 ± 67.2	1115.0 ± 145.1	2.8 ± 0.3	88.8 ± 9.2	143.3 ± 14.9	193.0 ± 20.7	506.4 ± 115.2.	<LOQ
**I**	10077.5 ± 1266.7	1345.5 ± 218.2	2760.0 ± 440.8	3.7 ± 0.5	51.0 ± 7.9	5.5 ± 1.1	0.2 ± 0.1	0.3 ± 0.1	108.4 ± 11.4
**J**	6759.0 ± 853.1	264.8 ± 53.8	1385.5 ± 139.9	1.6 ± 0.2	54.6 ± 3.5	4.6 ± 0.5	0.1 ± 0.1	0.9 ± 0.1	109.5 ± 16.2
**K**	8469.5 ± 1007.0	116.6 ± 15.6	1367.5 ± 233.4	1.2 ± 0.1	45.1 ± 2.3	7.1 ± 1.4	0.3 ± 0.1	0.2 ± 0.1	122.1 ± 13.1
**L**	11785.0 ± 1715.9	335.1 ± 40.6	1762.0 ± 238.6	9.7 ± 1.1	38.2 ± 2.2	8.2 ± 0.8	0.2 ± 0.1	0.8 ± 0.1	351.6 ± 46.3
**M**	12940.0 ± 2202.9	224.0 ± 24.9	1641.5 ± 293.6	2.2 ± 0.3	40.2 ± 2.7	7.2 ± 0.7	0.3 ± 0.1	0.4 ± 0.1	124.2 ± 15.5
**N**	7387.0 ± 820.7	565.4 ± 70.4	2005.5 ± 384.9	7.5 ± 0.7	53.5 ± 3.3	6.9 ± 0.7	0.1 ± 0.1	0.2 ± 0.1	142.5 ± 21.9
**O**	11710.0 ± 2036.4	198.1 ± 22.6	1588.5 ± 155.0	2.3 ± 0.3	35.2 ± 2.1	6.5 ± 0.7	0.2 ± 0.1	0.2 ± 0.1	159.6 ± 26.0
**P**	7220.0 ± 745.1	521.7 ± 58.4	2227.5 ± 304.9	2.7 ± 0.4	44.9 ± 3.7	7.6 ± 0.8	<LOQ	0.4 ± 0.1	256.3 ± 31.4
**Q**	5692.5 ± 884.6	112.7 ± 16.2	639.6 ± 88.3	3.5 ± 0.6	64.0 ± 3.3	4.4 ± 0.6	0.1 ± 0.1	0.3 ± 0.1	167.5 ± 27.9
**R**	7086.5 ± 1073.6	207.1 ± 34.7	1307.1 ± 241.0	2.4 ± 0.3	23.4 ± 1.1	7.2 ± 1.2	0.5 ± 0.1	0.2 ± 0.1	111.3 ± 12.2
**S**	5573.0 ± 669.9	289.3 ± 52.5	749.6 ± 125.2	13.8 ± 2.8	61.5 ± 1.8	6.9 ± 0.8	0.2 ± 0.1	0.2 ± 0.1	105.4 ± 23.4
**T**	8099.5 ± 852.9	1990.5 ± 224.0	1265.0 ± 275.0	2.7 ± 0.4	30.5 ± 2.6	7.9 ± 1.0	<LOQ	0.2 ± 0.1	104.1 ± 24.6
**U**	8318.5 ± 1461.7	139.9 ± 15.3	318.5 ± 46.6	7.5 ± 1.0	45.2 ± 4.1	2.2 ± 0.2	0.2 ± 0.1	<LOQ	145.7 ± 23.8
**V**	5736.5 ± 785.3	115.5 ± 21.1	1263.0 ± 180.4	8.4 ± 1.7	54.6 ± 3.6	5.4 ± 0.7	0.3 ± 0.1	0.4 ± 0.1	84.2 ± 10.2
**Sample**	***p*** **-Coumaric** **Acid**	**Ferulic** **Acid**	**Luteolin**	**Quercetin**	**Apigenin**	**Diosmetin**	**Kampferol**	**Protocatechuic Acid**	**Naringenin**
**A**	124.8 ± 16.8	5.1 ± 0.5	57.0 ± 6.1	35.4 ± 4.2	18.1 ± 2.5	25.0 ± 2.6	4.2 ± 0.5	1406.0 ± 159.7	41.5 ± 4.7
**B**	97.4 ± 10.7	8.7 ± 1.1	69.1 ± 7.6	330.2 ± 41.5	66.4 ± 7.0	130.4 ± 13,4	112.5 ± 16.6	1794.5 ± 226.3	40.2 ± 5.1
**C**	113.8 ± 16.7	18.0 ± 1.8	64.7 ± 10.0	331.6 ± 43.6	14.8 ± 1.8	46.7 ± 8.2	2.3 ± 0.3	1419.5 ± 184.5	34.9 ± 5.8
**D**	98.9 ± 11.1	24.3 ± 3.7	108.4 ± 13.5	75.2 ± 7.7	24.2 ± 3.7	16.9 ± 2.0	28.2 ± 3.8	853.9 ± 125.4	42.9 ± 5.6
**E**	102.2 ± 12.8	58.1 ± 6.0	332.9 ± 36.0	493.4 ± 58.3	21.5 ± 3.5	27.5 ± 2.8	165.1 ± 20.4	1311.5 ± 172.1	33.0 ± 6.1
**F**	142.5 ± 18.4	4.8 ± 0.5	21.7 ± 2.3	608.5 ± 112.0	25.0 ± 2.6	18.5 ± 2.8	18.2 ± 2.3	1237.5 ± 198.4	36.1 ± 4.2
**G**	72.7 ± 7.3	21.3 ± 2.8	93.1 ± 12.3	227.7 ± 25.1	16.3 ± 2.4	27.8 ± 3.1	26.9 ± 4.8	1023.5 ± 107.8	28.8 ± 3.2
**H**	112.5 ± 17.3	41.0 ± 7.0	106.9 ± 13.2	2015.5 ± 208.2	24.8 ± 3.2	35.3 ± 4.6	211.5 ± 24.2	1210.0 ± 161.3	48.2 ± 6.9
**I**	10.4 ± 1.2	7.0 ± 0.8	5.8 ± 0.7	44.9 ± 6.9	0.1 ± 0.1	10.4 ± 1.3	<LOQ	297.5 ± 36.0	48.0 ± 8.0
**J**	16.4 ± 1.8	3.8 ± 0.4	9.4 ± 1.0	22.5 ± 5.8	0.1 ± 0.1	16.9 ± 2.0	<LOQ	242.3 ± 29.6	37.2 ± 5.0
**K**	14.5 ± 2.0	7.7 ± 1.5	7.1 ± 0.9	10.6 ± 1.2	0.1 ± 0.1	5.3 ± 1.0	<LOQ	510.1 ± 74.3	37.8 ± 3.9
**L**	16.3 ± 1.7	6.8 ± 0.8	16.1 ± 1.8	280.5 ± 28.4	0.2 ± 0.1	22.0 ± 2.5	1.1 ± 0.1	534.3 ± 57.1	46.8 ± 8.5
**M**	16.6 ± 1.7	7.0 ± 0.7	10.0 ± 1.0	23.3 ± 1.8	0.1 ± 0.1	12.2 ± 1.5	0.3 ± 0.1	667.4 ± 82.4	52.5 ± 6.3
**N**	9.3 ± 1.5	5.0 ± 0.8	8.9 ± 1.1	30.3 ± 3.3	0.1 ± 0.1	11.1 ± 2.0	<LOQ	280.7 ± 38.3	50.2 ± 6.0
**O**	12.7 ± 1.6	6.1 ± 0.6	6.2 ± 1.1	21.8 ± 3.1	0.1 ± 0.1	9.5 ± 1.1	<LOQ	590.3 ± 129.4	35.4 ± 5.9
**P**	11.8 ± 1.5	7.8 ± 0.9	6.9 ± 1.0	193.7 ± 23.8	0.1 ± 0.1	11.8 ± 1.3	<LOQ	291.1 ± 36.6	61.5 ± 15.5
**Q**	8.5 ± 1.3	5.2 ± 0.6	8.0 ± 1.0	23.4 ± 3.2	0.1 ± 0.1	11.3 ± 2.1	<LOQ	303.2 ± 54.9	42.6 ± 6.8
**R**	9.2 ± 1.3	6.8 ± 0.9	7.8 ± 1.2	27.0 ± 4.1	0.1 ± 0.1	10.5 ± 1.5	<LOQ	322.5 ± 44.9	54.2 ± 6.3
**S**	9.2 ± 1.2	3.5 ± 0.4	7.9 ± 1.7	13.5 ± 2.0	0.1 ± 0.1	8.5 ± 1.0	<LOQ	285.7 ± 39.1	31.3 ± 3.8
**T**	7.6 ± 1.4	6.3 ± 0.9	3.5 ± 0.4	12.9 ± 1.8	0.1 ± 0.1	7.0 ± 0.9	<LOQ	388.7 ± 63.8	50.9 ± 5.5
**U**	10.5 ± 1.3	3.8 ± 0.5	6.8 ± 0.7	20.0 ± 2.4	0.1 ± 0.1	12.2 ± 1.4	0.2 ± 0.1	255.4 ± 38.9	32.9 ± 5.2
**V**	7.0 ± 0.8	6.4 ± 0.6	9.8 ± 1.5	284.2 ± 57.3	0.1 ± 0.1	17.3 ± 2.6	1.6 ± 0.1	305.4 ± 47.0	51.0 ± 5.9

**Table 5:** Inhibition halos measured for the extracts (10 mg/mL) assayed with different strains.

Extract	*S. aureus*	*S. epidermidis*	*S. pyogenes*	*E. faecalis*	*B. subtilis*	*B. cereus*	*P. aeruginosa*	*E. coli*	*C. albicans*
**A**	-	-	-	-	-	8	-	-	-
**B**	-	-	-	-	-	-	-	-	-
**C**	-	-	-	-	-	9	-	-	-
**D**	-	-	-	-	-	9	-	-	-
**E**	-	-	-	-	-	-	-	-	-
**F**	-	-	-	-	-	9	-	-	-
**G**	-	-	-	-	-	9	-	-	-
**H**	-	-	-	-	-	9	-	-	-
**I**	9	-	-	-	12	8	-	-	-
**J**	9	-	-	-	12	8	-	-	-
**K**	9	-	-	-	17	-	-	-	-
**L**	-	-	-	-	11	-	-	-	-
**M**	9	14	-	-	12	9	-	-	-
**N**	-	-	-	-	12	-	-	-	-
**O**	-	-	-	-	11	-	-	-	-
**P**	-	-	-	-	14	-	-	-	-
**Q**	-	11	-	-	13	-	-	-	-
**R**	-	-	-	-	13	-	-	-	-
**S**	-	-	-	-	8	-	-	-	-
**T**	-	-	-	-	10	-	-	-	-
**U**	-	-	-	-	10	-	-	-	-
**V**	-	-	-	-	8	-	-	-	-

**Table 6:** Strains used for the assay and growth conditions.

Strains	Growth Conditions
**Gram-positive bacteria** *Staphylococcus aureus* WDCM 00034	37 °C for 24 h
*Staphylococcus epidermidis* WDCM 00036	37 °C for 24 h
*Streptococcus pyogenes* ATCC 19615	37 °C for 24–48 h
*Enterococcus faecalis* WDCM 00087	37 °C for 24 h
*Bacillus subtilis* WDCM 00003	30 °C for 24 h
*Bacillus cereus* WDCM 00001	30 °C for 24 h
**Gram-negative bacteria**	
*Pseudomonas aeruginosa* WDCM 00025	25 °C for 24–48 h
*Escherichia coli* WDCM 00013	37 °C for 24 h
**Yeast**	
*Candida albicans* WDCM 00054	25 °C for 48–72 h

## Data Availability

Not applicable.

## Sample Availability

Samples of the compounds are available from the authors.

## References

[1] Bottone A., Cerulli A., Durso G., Masullo M., Montoro P., Napolitano A., Piacente S. (2019). Plant Specialized Metabolites in Hazelnut (Corylus avellana) Kernel and Byproducts: An Update on Chemistry, Biological Activity, and Analytical Aspects. Planta Med..

[2] Shahidi F., Alasalvar C., Liyana-Pathirana C.M. (2007). Antioxidant phytochemicals in hazelnut kernel (*Corylus avellana* L.) and hazelnut byproducts. J. Agric. Food Chem..

[3] Ramalhosa E., Delgado T., Estevinho L., Pereira J.A. (2011). Hazelnut (*Corylus avellana* L.) Cultivars and Antimicrobial Activity. Nuts and Seeds in Health and Disease Prevention.

[4] Ciemniewska-Zytkiewicz H., Verardo V., Pasini F., Bryś J., Koczoń P., Caboni M.F. (2015). Determination of lipid and phenolic fraction in two hazelnut (*Corylus avellana* L.) cultivars grown in Poland. Food Chem..

[5] Şenol H. (2019). Biogas potential of hazelnut shells and hazelnut wastes in Giresun City. Biotechnol. Reports.

[6] Demirbas A. (2006). Furfural production from fruit shells by acid-catalyzed hydrolysis. Energy Sources Part A Recover. Util. Environ. Eff..

[7] Fanali C., Gallo V., Posta S.D., Dugo L., Mazzeo L., Cocchi M., Piemonte V., De Gara L. (2021). Choline chloride–lactic acid-based NADES as an extraction medium in a response surface methodology-optimized method for the extraction of phenolic compounds from hazelnut skin. Molecules.

[8] Kumar A., Kumar P., Koundal R., Agnihotri V.K. (2016). Antioxidant properties and UPLC–MS/MS profiling of phenolics in jacquemont’s hazelnut kernels (Corylus jacquemontii) and its byproducts from western Himalaya. J. Food Sci. Technol..

[9] Rusu M.E., Fizeșan I., Pop A., Gheldiu A.M., Mocan A., Crișan G., Vlase L., Loghin F., Popa D.S., Tomuta I. (2019). Enhanced recovery of antioxidant compounds from hazelnut (*Corylus avellana* L.) involucre based on extraction optimization: Phytochemical profile and biological activities. Antioxidants.

[10] Shahidi F., Ambigaipalan P. (2015). Phenolics and polyphenolics in foods, beverages and spices: Antioxidant activity and health effects-A review. J. Funct. Foods.

[11] Herrera R., Hemming J., Smeds A., Gordobil O., Willför S., Labidi J. (2020). Recovery of bioactive compounds from hazelnuts and walnuts shells: Quantitative–qualitative analysis and chromatographic purification. Biomolecules.

[12] Barbulova A., Colucci G., Apone F. (2015). New trends in cosmetics: By-products of plant origin and their potential use as cosmetic active ingredients. Cosmetics.

[13] Pagano C., Marinozzi M., Baiocchi C., Beccari T., Calarco P., Ceccarini M.R., Chielli M., Orabona C., Orecchini E., Ortenzi R. (2020). Bioadhesive Polymeric Films Based on Red Onion Skins Extract for Wound Treatment: An Innovative and Eco-Friendly Formulation. Molecules.

[14] Pagano C., Perioli L., Baiocchi C., Bartoccini A., Beccari T., Blasi F., Calarco P., Ceccarini M.R., Cossignani L., di Michele A. (2020). Preparation and characterization of polymeric microparticles loaded with Moringa oleifera leaf extract for exuding wound treatment. Int. J. Pharm..

[15] Pagano C., Baiocchi C., Beccari T., Blasi F., Cossignani L., Ceccarini M.R., Orabona C., Orecchini E., Di Raimo E., Primavilla S. (2021). Emulgel loaded with flaxseed extracts as new therapeutic approach in wound treatment. Pharmaceutics.

[16] Yuan B., Lu M., Eskridge K.M., Hanna M.A. (2018). Valorization of hazelnut shells into natural antioxidants by ultrasound-assisted extraction: Process optimization and phenolic composition identification. J. Food Process. Eng..

[17] (2004). Encyclopedia Britannica online. Choice Rev. Online.

[18] Dzah C.S., Duan Y., Zhang H., Wen C., Zhang J., Chen G., Ma H. (2020). The effects of ultrasound assisted extraction on yield, antioxidant, anticancer and antimicrobial activity of polyphenol extracts: A review. Food Biosci..

[19] Minatel I.O., Borges C.V., Ferreira M.I., Gomez H.A.G., Chen C.-Y.O., Lima G.P.P. (2017). Phenolic Compounds: Functional Properties, Impact of Processing and Bioavailability. Phenolic Compd. Biol. Act..

[20] Yuan B., Lu M., Eskridge K.M., Isom L.D., Hanna M.A. (2018). Extraction, identification, and quantification of antioxidant phenolics from hazelnut (Corylus avellana L.) shells. Food Chem..

[21] Masullo M., Cerulli A., Mari A., de Souza Santos C.C., Pizza C., Piacente S. (2017). LC-MS profiling highlights hazelnut (Nocciola di Giffoni PGI) shells as a byproduct rich in antioxidant phenolics. Food Res. Int..

[22] Pérez-Armada L., Rivas S., González B., Moure A. (2019). Extraction of phenolic compounds from hazelnut shells by green processes. J. Food Eng..

[23] Xu Y., Sismour E.N., Parry J., Hanna M.A., Li H. (2012). Nutritional composition and antioxidant activity in hazelnut shells from US-grown cultivars. Int. J. Food Sci. Technol..

[24] Paixão N., Perestrelo R., Marques J.C., Câmara J.S. (2007). Relationship between antioxidant capacity and total phenolic content of red, rosé and white wines. Food Chem..

[25] Dunnill C., Patton T., Brennan J., Barrett J., Dryden M., Cooke J., Leaper D., Georgopoulos N.T. (2017). Reactive oxygen species (ROS) and wound healing: The functional role of ROS and emerging ROS-modulating technologies for augmentation of the healing process. Int. Wound J..

[26] Nazzaro M., Valentina Mottola M., La Cara F., Del Monaco G., Patrizia Aquino R., Volpe M.G. (2012). Extraction and characterization of biomolecules from agricultural wastes. Chem. Eng. Trans..

[27] Perva-Uzunalić A., Škerget M., Knez Ž., Weinreich B., Otto F., Grüner S. (2006). Extraction of active ingredients from green tea (Camellia sinensis): Extraction efficiency of major catechins and caffeine. Food Chem..

[28] Oliveira I., Sousa A., Morais J.S., Ferreira I.C.F.R., Bento A., Estevinho L., Pereira J.A. (2008). Chemical composition, and antioxidant and antimicrobial activities of three hazelnut (Corylus avellana L.) cultivars. Food Chem. Toxicol..

[29] Kubo I., Fujita K.I., Nihei K.I., Nihei A. (2004). Antibacterial Activity of Akyl Gallates against Bacillus subtilis. J. Agric. Food Chem..

[30] Proestos C., Chorianopoulos N., Nychas G.J.E., Komaitis M. (2005). RP-HPLC analysis of the phenolic compounds of plant extracts. Investigation of their antioxidant capacity and antimicrobial activity. J. Agric. Food Chem..

[31] Roila R., Ranucci D., Valiani A., Galarini R., Servili M., Branciari R. (2019). Antimicrobial and anti-biofilm activity of olive oil by-products against Campylobacter spp. isolated from chicken meat. Acta Sci. Pol. Technol. Aliment..

[32] Roila R., Valiani A., Ranucci D., Ortenzi R., Servili M., Veneziani G., Branciari R. (2019). Antimicrobial efficacy of a polyphenolic extract from olive oil by-product against “Fior di latte” cheese spoilage bacteria. Int. J. Food Microbiol..

[33] Kayumov A.R., Khakimullina E.N., Sharafutdinov I.S., Trizna E.Y., Latypova L.Z., Thi Lien H., Margulis A.B., Bogachev M.I., Kurbangalieva A.R. (2015). Inhibition of biofilm formation in Bacillus subtilis by new halogenated furanones. J. Antibiot..

[34] Giacometti A., Cirioni O., Schimizzi A.M., Del Prete M.S., Barchiesi F., D’Errico M.M., Petrelli E., Scalise G. (2000). Epidemiology and microbiology of surgical wound infections. J. Clin. Microbiol..

[35] Ronco T., Aragao M.F., Svenningsen S., Christensen J.B., Permin A., Saaby L., Bionda N., Lantz E.E., Olsen R.H. (2021). Efficacy of a novel antimicrobial hydrogel for eradication of Staphylococcus epidermidis, Staphylococcus aureus and Cutibacterium acnes from preformed biofilm and treatment performance in an in vivo MRSA wound model. JAC-Antimicrobial Resist..

[36] Pîrvǎnescu H., BǎlǎŞoiu M., Ciurea M.E., Bǎlǎşoiu A.T., Mǎnescu R. (2014). Wound infections with multi-drug resistant bacteria. Chir..

[37] Bottone E.J. (2010). Bacillus cereus, a volatile human pathogen. Clin. Microbiol. Rev..

[38] Pagano C., Perioli L., Blasi F., Bastianini M., Chiesi C., Cossignani L. (2017). Optimisation of phenol extraction from wine using layered double hydroxides and technological evaluation of the bioactive-rich powder. Int. J. Food Sci. Technol..

[39] Rocchetti G., Pagnossa J.P., Blasi F., Cossignani L., Piccoli R.H., Zengin G., Montesano D., Cocconcelli P.S., Lucini L. (2020). Phenolic profiling and in vitro bioactivity of Moringa oleifera leaves as affected by different extraction solvents. Food Res. Int..

[40] Ianni F., Blasi F., Angelini P., Di Simone S.C., Flores G.A., Cossignani L., Venanzoni R. (2021). Extraction optimization by experimental design of bioactives from pleurotus ostreatus and evaluation of antioxidant and antimicrobial activities. Processes.

[41] Oliva E., Viteritti E., Fanti F., Eugelio F., Pepe A., Palmieri S., Sergi M., Compagnone D. (2021). Targeted and semi-untargeted determination of phenolic compounds in plant matrices by high performance liquid chromatography-tandem mass spectrometry. J. Chromatogr. A.

[42] Balouiri M., Sadiki M., Ibnsouda S.K. (2016). Methods for in vitro evaluating antimicrobial activity: A review. J. Pharm. Anal..

